# A Pervasive Respiratory Monitoring Sensor for COVID-19 Pandemic

**DOI:** 10.1109/OJEMB.2020.3042051

**Published:** 2020-12-02

**Authors:** Xiaoshuai Chen, Shuo Jiang, Zeyu Li, Benny Lo

**Affiliations:** Hamlyn CentreImperial College London 4615 London SW7 2AZ U.K.; College of Electronics and Information EngineeringTongji University 12476 Shanghai 201804 China; Department of Electrical and Computer EngineeringDuke University 3065 Durham NC 27708 USA

**Keywords:** Ambient sensor, cough detection, COVID-19 pandemic, healthcare, respiration

## Abstract

*Goal:* The SARS-CoV-2 viral infection could cause severe acute respiratory syndrome, disturbing the regular breathing and leading to continuous coughing. Automatic respiration monitoring systems could provide the necessary metrics and warnings for timely intervention, especially for those with mild symptoms. Current respiration detection systems are expensive and too obtrusive for any large-scale deployment. Thus, a low-cost pervasive ambient sensor is proposed. *Methods:* We will posit a barometer on the working desk and develop a novel signal processing algorithm with a sparsity-based filter to remove the similar-frequency noise. Three modes (coughing, breathing and others) will be conducted to detect coughing and estimate different respiration rates. *Results:* The proposed system achieved 97.33% accuracy of cough detection and 98.98% specificity of respiration rate estimation. *Conclusions:* This system could be used as an effective screening tool for detecting subjects suffering from COVID-19 symptoms and enable large scale monitoring of patients diagnosed with or recovering.

## I. Introduction

Coronavirus disease 2019 (COVID-19) has emerged as a pandemic and it has affected over 210 countries. More than 42 million people are infected globally, and the number of cases is increasing rapidly. The demand for healthcare systems worldwide has risen abruptly and caused significant disruptions to the delivery of care. Abnormal respiration is one of the most obvious symptoms of SARS-CoV-2 viral infection. COVID-19 symptoms are fever (83%–99%), dry cough (59.4%–82%) and respiratory distress (∼55%) [1]. Abnormal respiration rate is also one of the indications of the severity of a COVID-19 patient's condition and it is often used as an index to support clinical decisions for timely interventions at the inchoate stage of the infection. Respiration involves a complex biological interaction between the central nervous system, respiratory-related motor neurons, and respiration muscles [2]. Based on these interactions, various techniques have been developed for respiratory rate detection. However, accurate, continuous, and pervasive respiratory rate measurement is challenging and still under investigation, especially for direct measurement. Ding and Liu *et al.* [3], [4] have recently reviewed the current wearable sensing and telehealth technologies, and in general, the respiratory rate (RR) is captured in the following three ways:

### A. Respiratory Airflow Detection

Respiratory airflow could induce variation in pressure, temperature, humidity and acoustic in the vicinity of subjects [5]. One of the standard clinical devices for respiration monitoring is the Spirometer, based on a pneumotachograph measuring the volume of air inhaled and exhaled by a patient in each breath and the time it takes for each breath [6]. Joseph M *et al.* [7] introduced a nasal prong method connected to a pressure transducer to measure nasal flow in sleep apnea and hypopnea patients. Capnography is another clinic respiration measurement that detects the End-Tidal Carbon Dioxide levels for hypoventilation [8], integrating with a face mask to measure the $C{O_2}$ concentration in the exhaled air [9]. However, it is expensive and could cause difficulty in breathing. Das *et al.* [10] proposed a monitoring system based on BTC (Negative Temperature Coefficient) type thermistor and the thermistor placed in the nasal or oral position, detecting air temperature fluctuations during inhalation and exhalation. Thermal imaging [11] captured by infrared cameras is a non-contact method to estimate the RR from thermal images through detecting the air temperature variations near the limited nose or mouth area. Humidity gradient can also be used to detect respiration. Kano *et al.* [12] embedded a $Si{O_2}$ nanoparticle thin film on a flexible substrate onto a sensor chip, detecting the humidity variations and measuring a subject's respiration rate. Cheng *et al.* [13] proposed a polymer film sensor using 1D nanowires to measure the water molecules' absorption for estimating the RR. The performance of thermal and humidity gradient sensing greatly depends on the environment. Acoustic sensing methods could achieve high accuracy in RR detection by capturing the acoustic signal induced by breathing where sensors are positioned at the nasal and tracheal locations or ear canal [14], [15]. However, imaging and acoustic sensing could lead to privacy concerns.

### B. Abdominal Volume Changes and Body Movements

Sensing respiratory-related physical activities such as abdominal or chest movements is another common approach. Bates *et al.* [16] demonstrated a tri-axial accelerometer-based method for detecting the breathing motions. Zhang *et al.* [17] proposed a waist-worn device based on a triboelectric nanogenerator (TENG) for sensing breathing, as the breathing process is carried out by contraction and relaxation of the diaphragm and which causes the subtle variation of the abdominal circumference. Different modes of respiration, such as abdominal and thoracic respiration, and various daily activities (standing, lying and sitting), were assessed by the TENG sensor system. Another stretchable and wearable technology based on an inkjet-printed strain gauge sensor was developed in [18]. Huang *et al.* [19] proposed a non-invasive respiratory monitoring system by integrating an array of load sensors in the e-textile bed sheet to detect pressure variance in the breathing cycle. However, the power supply and the sensors' life cycles are limited, and other body movements will induce significant noise and severely affect the detection results.

### C. Physiological Signals

Respiratory signals, can be extracted from cardiovascular measurements, such as the electrocardiogram (ECG) and the photoplethysmography (PPG) signals. O'Brien *et al.* [20] showed that the respiratory signal could be extracted from a single lead ECG sensor signal. The resulting respiratory signal has a correlation coefficient of around 0.75, with measurements from inductance plethysmography. RR measurement from a single lead ECG shows better results than methods based on the mean electrical axis while it can classify epochs of disordered respiration during sleeping with 82% accuracy. Meredith *et al.* [21] reviewed PPG methods for RR monitoring. PPG based methods are shown to be more robust and reliable in RR measurement compare to ECG based methods. However, both ECG and PPG would require the patient to wear the sensor, and user compliance would be a major issue for large scale deployment, and the sensors are prone to motion artifacts.

This paper proposes an ambient sensing system for respiration monitoring. This system is based on a single barometric sensor positioned in a working or living environment. In particular, the miniaturized sensor can be placed on the desk and capture the user's respiration and coughing pervasively. The system was conducted based on the hypothesis that respiration airflow will cause subtle air pressure variation in close proximity, and highly sensitive barometric sensors can detect such small changes. This small sensor can be built as low-cost devices for large scale deployment and potentially be used to monitor patients suffering from COVID-19 symptoms as a screening tool for detecting early signs of infection.

## II. Materials and Methods

### A. The Ambient System Setup

**Fig. 1.**
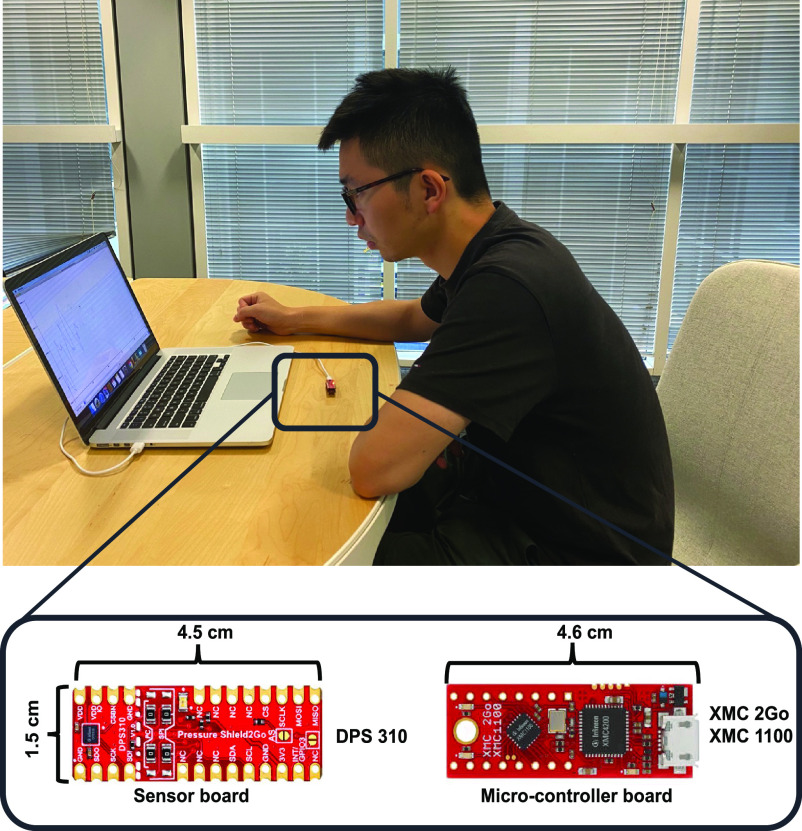
The respiration system which is based on a single barometer located on the working desk and connected to a personal computer.

The ambient sensor system was designed based on a single barometric sensor (DPS310, Infineon, German). The barometric sensor has a pressure precision of ± 0.002hPa and an operation range of 300--1200 hPa. The sensor prototype measures 3 cm × 2 cm. It was controlled by the microcontroller (XMC2Go, Infineon, German) and it is designed on the desk (Fig. 1). The working desk is located in an indoor environment, such as the living room or the personal office, with slow and constant airflow. The barometric sensor is connected to a personal computer, but it can be extended to the wireless sensor.

### B. Algorithm Design

**Fig. 2.**
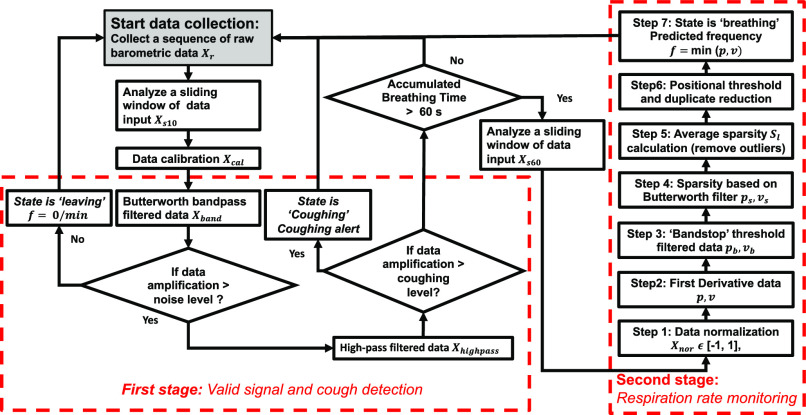
The general algorithm flowchart for respiration monitoring, including valid signal detection, coughing event detection and different respiration rate estimation.

Despite the barometric sensor's high sensitivity, the barometric signal is very noisy, and it requires a robust signal processing algorithm to extract the respiration information. A lightweight decision tree algorithm is designed and developed with the aim of enabling on-node processing, and the flowchart of the algorithm is illustrated in Fig. 2. Three modes are designed in this system: coughing, breathing and others (randomly talking or hold breathing).

#### 1) Valid Signal and Coughing Detection Stage

**Fig. 3.**
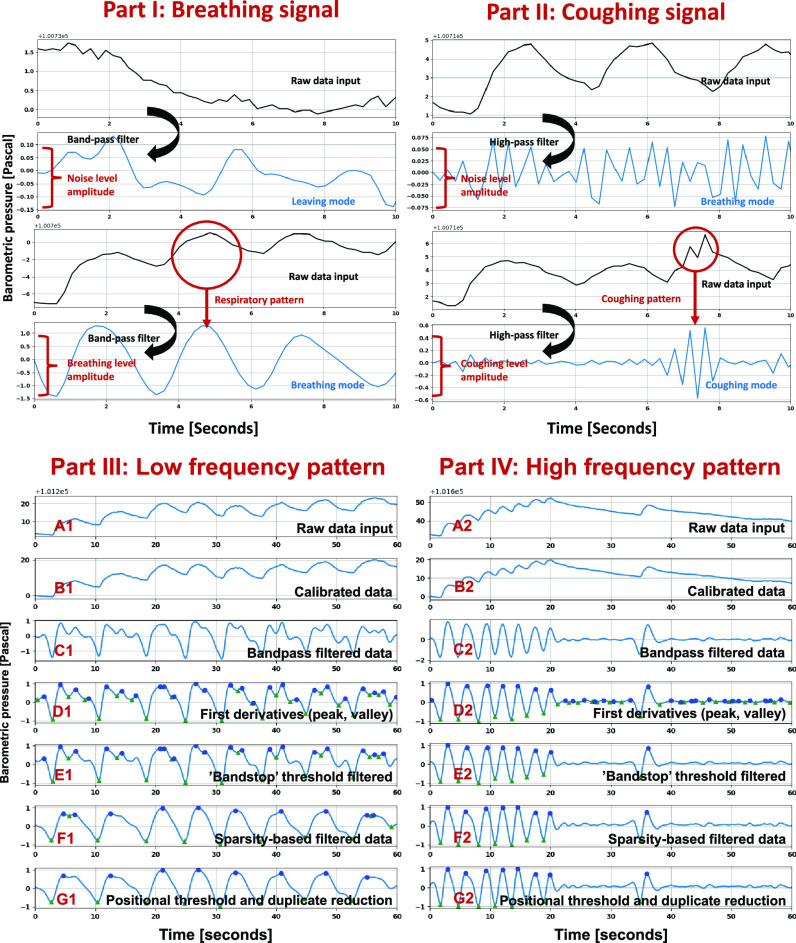
(Part I) Breathing defection stage. (Part II) Coughing signal detection. (Part III) Low-frequency signal processing. (Part IV) High-frequency signal processing.

The first stage of the decision tree algorithm is to distinguish ‘breathing’ from ‘others’ (Fig. 3. Part I). The barometric sensor has the sensing precision of ± 0.002hPa (0.2Pa), a reference to the noise level ${{\rm{A}}_{{\rm{\rm noise}}}}$. If the data amplitude is lower than the noise level, this data sequence will be classified as the ‘others’. Then the system will continue to collect the next data stream. Once this collected signal is over the noise level, a high-pass filter $[ {{\rm{\ }}{{\rm{f}}_{{\rm{highcut}}}}} ]$ will be applied to raw data input. The coughing signal has a similar frequency to the noise and a similar amplitude to the breathing signal. A coughing threshold was selected empirically based on the different coughing test patterns (Fig. 3. Part II).

The second stage of the algorithm detects coughing from the ‘breathing’ mode. If the high pass filtered signal level ${{\rm{A}}_{{\rm{highpass}}}}$ is larger than the coughing level threshold ${{\rm{T}}_{{\rm{coughing}}}}$, the system will be switched to the ‘coughing’ mode and counting the frequency and incidence of coughing. If high pass filtered data is lower than the coughing level, the system status will remain in the ‘breathing’ mode. Once the accumulated breathing time is equal to or larger than the 60s, the signals will then be processed to detect breathing and coughing.

#### 2) Respiration Rate Monitoring Stage

The data output from the bandpass Butterworth filtered signal may show different amplitude because of the different breathing strengths each time. In the first step, a data normalization scaling into a standard form ${\rm{X}}{{\rm{\ }}_{{\rm{nor}}}}$ $ \in $[ −1, 1] is introduced to enhance the detection of breathing (Fig. 3. Part III. C1 and Part IV. C2). First derivative is used in the second step to extract the peak and valley points of the signals caused by the inhalation and exhalation process for estimating breathing rates.

 Although the bandpass filterer can reduce the low and high-frequency noise, there still a small amplitude noise with a similar frequency to the signal (Fig. 3. Part III. D1 and Part IV. D2). This kind of noise has a lower amplitude than the standard signal amplitude and is located near the zero-crossing area. In the third step, a double direction ‘bandstop’ threshold [${T_{\rm low}},\ {T_{\rm high}}$ ] can reduce the zero-crossing noise. This ‘bandstop’ threshold will remove all the features between a designed low threshold ${T_{\rm low}}$ and a high threshold ${T_{\rm high}}$.

The signal processing is mainly divided into the low-frequency group (Fig. 3. Part III) and the high-frequency group (Fig. 3. Part IV). Bandstop filter can effectively remove the subtle noise near the zero-crossing point of the high-frequency groups (Fig. 3. Part IV. E2) but cannot reduce the local noise with a relatively large amplitude close to the signal points in the low-frequency groups (Fig. 3. Part III. E1). A low-frequency breathing pattern may contain high-frequency noise, which is close to a high-frequency signal band. Thus, an additional low pass filter is applied to the lower frequency groups. Considering breathing signals may vary with different subjects, it is essential to set the correlated Butterworth filter to classify different breathing patterns of this situation.

**Fig. 4.**
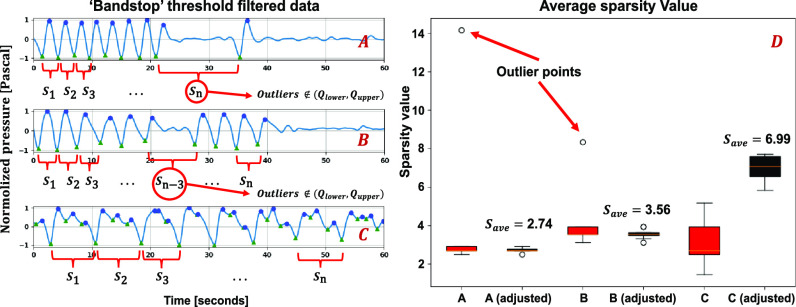
Local sparsity in different respiration patterns. (A) A higher breathing frequency at the beginning and the breathing was paused for a short period (Sn is detected as an outlier). (B) A slightly lower breathing frequency at the beginning, then the breathing was paused for a short time and breathing start again several times and finally stopped (Sn-3 is detected as an outlier). (C) Deep breathing with a large sparsity value. (D) shows sparsity value and the adjusted sparsity value by removing the outlier points (responding to A, B, C). The red box represents the raw average sparsity with all peak and valley points. The Black box represents the adjusted average sparsity value after removing the outliers from the raw average sparsity value.

This research proposes a novel sparsity-based method to set the Butterworth filter, which can classify different breathing patterns. Step four of the algorithm is to determine the average sparsity value calculation. A local sparsity value is defined as the distance between the two closest valleys (Fig. 4. A, B and C). The total sparsity of a respiratory pattern is the sum of all its local sparsity values $\{ {S_{1}},{S_{1}}, \ldots,{S_{\rm{n}}}\} $ detected. Average sparsity $\bar{S}$ should indicate the general breathing distribution without any outliers $\{ {{S_o}_1,\ {S_o}_1,\ {S_o}_1,\ \ \ldots \,\ {S_o}_m\ } \}\ $(Fig. 4. A and B). Still, it does not indicate the general breathing frequency. The average sparsity can be calculated in Eq. 1.

\begin{equation*}
\bar{s} = \frac{{(\mathop \sum \nolimits_{i = 1}^{n - m} {S_{\rm{i}}} - \mathop \sum \nolimits_{j = 1}^m {S_{\rm{o}}}_j)}}{{n - m}}\tag{1}
\end{equation*}

For any sparsity points, if it meets the relations:${S_o}\ \notin ({{F_{\rm lower}},{F_{\rm upper}}})$, it will be detected as an outlier point. Fig. 4. D. has shown the sparsity distribution for different situations. Then the algorithm uses the average sparsity to set up different Butterworth filters (Fig. 3. Part III. E1, F1 and Part IV. E2. F2). For the situation in Fig. 3. Part III. E1, which has a relatively large sparsity value containing much local noise at high-frequency bands, the signal will be processed with an additional low-pass filter. For the situations in Fig. 3. Part IV. E2, where the signal has a relatively low sparsity value, will be processed by an additional high-pass filter. Thus, based on the sparsity of the signal, a different filter is used.

The cutoff threshold is empirically selected for additional sparsity-based Butterworth filter (low-pass and high-pass filter). It will generate different values depending on different sparsity values generated in the last stage and it is shown as Eq. 2 and 3.

\begin{align*}
&Cutof{f_{\rm low}} = \frac{\alpha }{{{{\bar{S}}^2}}}\tag{2}\\
&Cutof{f_{\rm high}} = \frac{{{\rm{\ }}\bar{S}}}{\beta }\tag{3}
\end{align*}

where $\alpha $, $\beta $ are system parameters (set empirically), $\bar{S}$ is the average sparsity.

Algorithm: **Sparsity Based Filter.**
**Input:** Respiration pattern matrix $\{ {{X_1},\ {X_2},{X_3}, \ldots,{X_n}} \}$
**Output:** Sparsity filtered based respiration pattern matrix $\{ {{X_{{s_1}}},\ {X_{{s_2}}},{X_{{s_3}}}, \ldots,{X_{{s_n}}}} \}$
1: **while** i < n **do**
2: ${V_i} = \ Derivetives\ [{X_i}]$;
3:
${V_i} = \ {V_i}[{V_i} < {T_{lowerValley}}]$

4: **for** j in range(len(${V_i}$)-1) **do**
5: ${S_{temp}} = {V_{i,\ j + 1}} - {V_{i,\ j}}$;
6:
${S_{tot\_i}}.append({{S_{temp}}})$

7:
${\rm{Outliers}} \notin ({{F_{\rm lower}},\ {F_{\rm upper}}\ })$

8:
${\overline {\ S} _i} = ave[ {{S_{tot\_i}}.{\rm{\ Del\ }}[ {{\rm{Outliers}}} ]} ]$

9: **if**${\overline {\ S} _i} \ge {T_{filter}}$:
10: ${X_{s\_i}} = {\rm{\ \ }}LowpassFilter\ ({X_{\rm{i}}},{\rm{\ cutoff}} = {\rm{\ }}\frac{\alpha }{{{{\overline {\ S} }_i}^2}})$; i+=1;
11: **else:**
12: ${X_{s\_i}} = {\rm{\ \ }}HighpassFilter\ ({X_{\rm{i}}},{\rm{\ cutoff}} = {\rm{\ }}\frac{{{{\overline {\ S} }_i}}}{\beta })$; i+=1;
13: **return** $\{ {{X_{{s_1}}},\ {X_{{s_2}}},{X_{{s_3}}}, \ldots,{X_{{s_n}}}} \}$

The pseudocode in supplementary material shows the details of the algorithm for the sparsity-based filter. Although the local noise has been shrunk significantly in step five, small local noise still exists. Step six reduces the duplicated points such as peak-peak and valley-valley induced after thresholding (Fig. 3. Part III. F1). The final step is the respiration rate estimation.

### C. Spatial Sensitivity Study

The relative distance of sensors and users is an essential evaluation of the respiration monitoring system. Thus, an extensive study of analyzing the spatial sensitivity of the sensor is conducted in this research. For a relative spatial location [x, y, z] generated by the respiration signal, the signal energy can be extracted from a series of signal data. The valid energy ${E_{\rm valid}}$ can be obtained by integrating the average noise threshold level ${A_{\rm noise}}$ between a sliding window $[ {{t_1},\ {t_2}} ]$ with a respiration frequency $f$ shown in Eq. 4. If the respiration energy is larger than the valid level, it can be determined as a valid sensor location. If not, this will be determined as an invalid location.

\begin{equation*}
{E_{\rm valid}} = \mathop \int_{t1}^{t2} {A_{\rm noise}} * Sin\left({\frac{1}{f}} \right)dt\tag{4}
\end{equation*}

Based on the preliminary data (processing result from the bandpass filter), we investigated the sensor's effective sensing range on detecting the respiration rate (16 times/mins). We set up the working space with the relative horizontal offset of X from −10 cm to 10 cm (tangential to the user's orientation), Y from 0-25 cm (along to the user's direction) vertical offset from 25 cm to 35 cm. The nasal location is (0, 0, Z), while Z is chosen by 25cm, 30 cm, 35 cm.

### D. Experimental Protocol

A respiration monitoring protocol (Table I) is designed with the respiration rate monitoring part (E1-E3) and the cough detection part (E4-E6). In our research, 10 healthy subjects were recruited with consent (Table I). Each subject was required to follow the six groups (E1-E6) of our protocol to simulate different respiration patterns.

**TABLE I:** Experimental Protocol and Study Participants Characteristics

**Respiration protocols**		**Trials**
E1	Low Respiration Rate (1-11)	88
E2	Normal Respiration Rate (12-16)	40
E3	Fast Respiration Rate (17-20)	32
E4	Respiration Rate (Random)	100
E5	Coughing	100
E6	Others (talking or hold breathing)	100
**Characteristics**		**Testing set**
No. Subjects		10
Age (years)a		24 ± 2
Sex (No.)		M (6), F (4)
Height (cm)a		172 ± 9
Weight (kg)a		65 ± 12

^a^Data are expressed as mean ± std, dev.

## III. Results

**Fig. 5.**
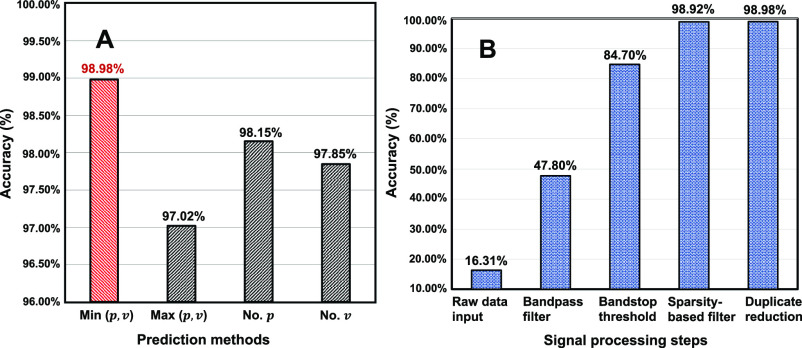
(A) Compared the different prediction methods based on peak and valley points. (the minimal number, the maximal number between peak and valley points, number of peak points, number of valley points). (B) Shows the accuracy improvements during different signal processing steps.

**Fig. 6.**
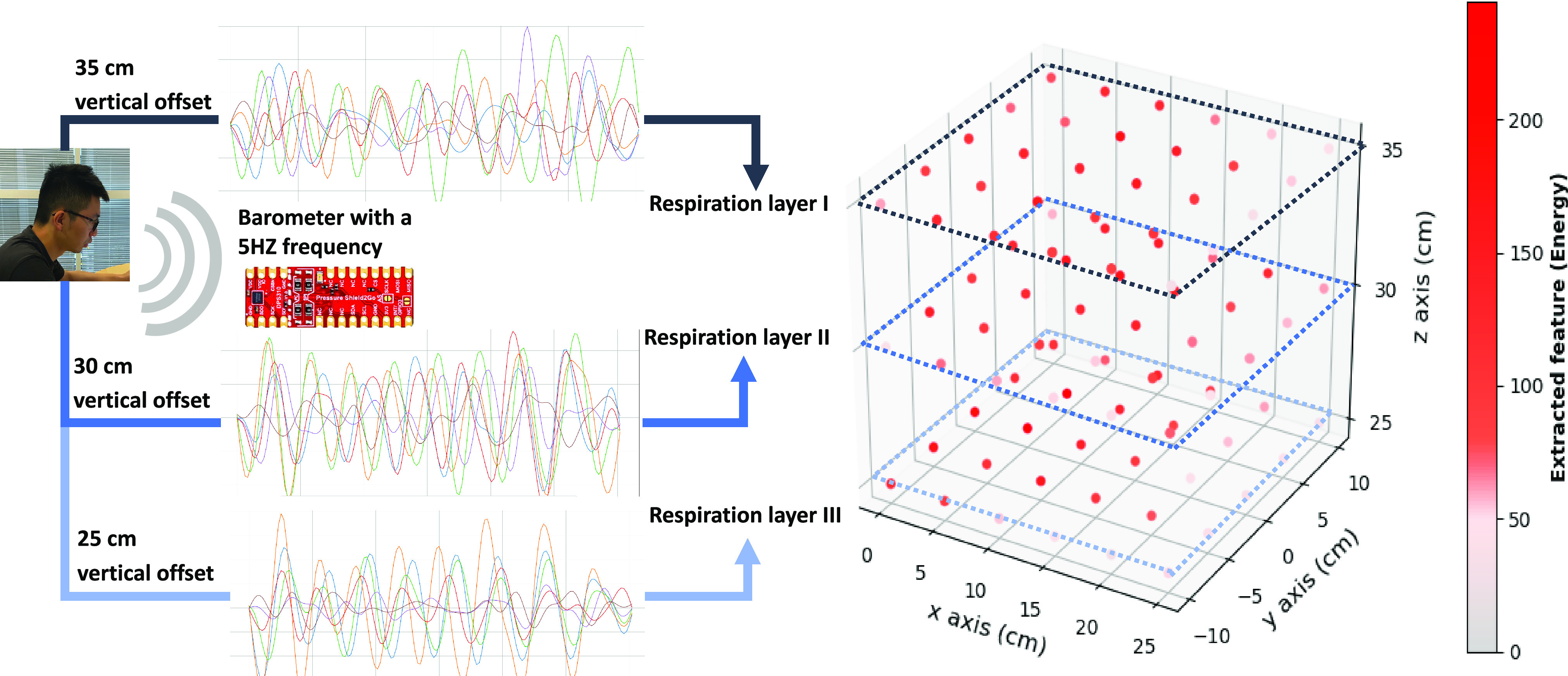
Spatial effects on respiration signal with the energy index. The user was breathing naturally (with a breathing rate around 16 times/min).

The cough detection data set contains 100 coughing patterns, 100 breathing patterns, and 100 other patterns from 10 subjects. The precision of coughing detection is 96%, recall is 99%, f1-score is 97% and support is 97 (events). The general detection accuracy is 97.33% (Confusion Matrix is shown in supplementary materials). For coughing detection, a few methods were used to captured the peak and valley points. The comparison results are shown in Fig. 5. The results show that the minimal number of peak and valley points has better accuracy than other methods before the sparsity-based filter. The sparsity-based filter has greatly improved the whole system performance and significantly enhanced robustness in different situations. The minimal number of the peak and valley points shows the best results with a general specificity of 98.98 % and the error proportion of E1 is 29.41%, E2 is 47.06% and E3 is 23.53%. In the sensitivity study, the sensor signal strength with an X offset of 25 cm, Y offset of 20 cm, Z offset of 30 cm is shown in Fig. 6. The signal strength was strong from 0-15 offset but became unstable when the distance is more than 20 cm.

## IV. Discussion

This system shows that the barometers can detect breathing and cough reliably in a working desk environment even if there are other people in the same room, as long as the others are at least 35 cm away from the sensor. A comparison of different respiration sensing systems is conducted in terms of specificity, system sensitivity, payload convenience, equipment expense, and privacy (Supplementary Material). Our system outperforms other systems with a specificity of 98.98% respiration rate monitoring. One potential limitation of the proposed system is that it is based on a relatively constant environment without any window or door opening/closing events during the monitoring process, as those events may cause a sudden increase/decrease of the room air pressure and thus affect the sensing accuracy. Arm movements may also affect the airflow diffusion temporarily if it moves over the sensors. Besides, the body's movements and orientations may result in different sensing areas; thus, a more robust system could be built with multi-barometer sensing arrays on the desk or integrated as badges or buttons to be worn on the chest. A patient study should be conducted to validate the proposed. With the development of the advanced barometric sensors, the device could be extended to quantify respiration in sleep, leading to other applications, such as sleep apnea detection, etc.

## V. Conclusion

This paper proposes using a barometric sensor as an ambient sensor for respiration rate estimation and coughing detection. From the experiments, we have shown the sensitivity of the sensor in capturing the subtle airflow variation caused by breathing and coughing in a desk working scenario. A lightweight signal processing algorithm is designed and proposed for detecting breathing and coughing. In addition, a sparsity-based filter is proposed for reducing the local high-frequency noise without attenuating or rejecting the coughing or high-frequency breathing signals. The experimental results of cough detection and respiration monitoring show the robustness and accuracy of the proposed method. The low cost and miniaturized sensor can be deployed as a pre-scanning tool for detecting people with COVID-19 symptoms and analyze the severity of the infection.

## VI. Supplementary Material

The supplementary materials (S. M) include the respiration signal strength distribution with the barometric sensor at different spatial locations (S. M: Fig. 1-5), confusion matrix for coughing detection (S. M: Fig. 6), the pseudocode of duplicate points reduction (S. M: Table. 1), a performance comparison of current respiration monitoring systems (S. M: Table. 2).

 the respiration signal strength distribution with the barometric sensor at different spatial locations (S. M: Figs. 1-5), confusion matrix for coughing detection (S. M: Fig. 6), the pseudocode of duplicate points reduction (S. M: Table. 1), a performance comparison of current respiration monitoring systems (S. M: Table. 2).
