# Supplementary material for: A Pervasive Respiratory Monitoring Sensor for COVID-19 Pandemic
Source: IEEE Open J Eng Med Biol. 2020 Dec 2;2:11–6. doi: 10.1109/OJEMB.2020.3042051 (PMC8545027; doi:10.1109/OJEMB.2020.3042051)
Supplement: the respiration signal strength distribution with the barometric sensor at different spatial locations (S. M: Figs. 1-5), confusion matrix for coughing detection (S. M: Fig. 6), the pseudocode of duplicate points reduction (S. M: Table. 1), a performance comparison of current respiration monitoring  [file supp1-3042051.pdf]

Supplementary Materials

A Pervasive Respiratory Monitoring Sensor for COVID-19 Pandemic

Xiaoshuai Chen, *Student Member, IEEE*, Shuo Jiang, Zeyu Li, Benny Lo*, *Senior Member, IEEE*

1. Description

The supplementary materials aim to provide extra information about our original work for COVID-19 Pandemic monitoring, which is useful for understanding our work and further improvements of related systems. It includes comprehensive performance testing, algorithm designing, prediction results, and system comparison. Four main sections are conducted, including the respiration signal strength distribution with the barometric sensor at different spatial locations (Fig. S1-S5), the confusion matrix for coughing detection (Fig. S6), the pseudocode of duplicate points reduction (Table. 1), a performance comparison of current respiration monitoring systems (Table. 2).

2. Details

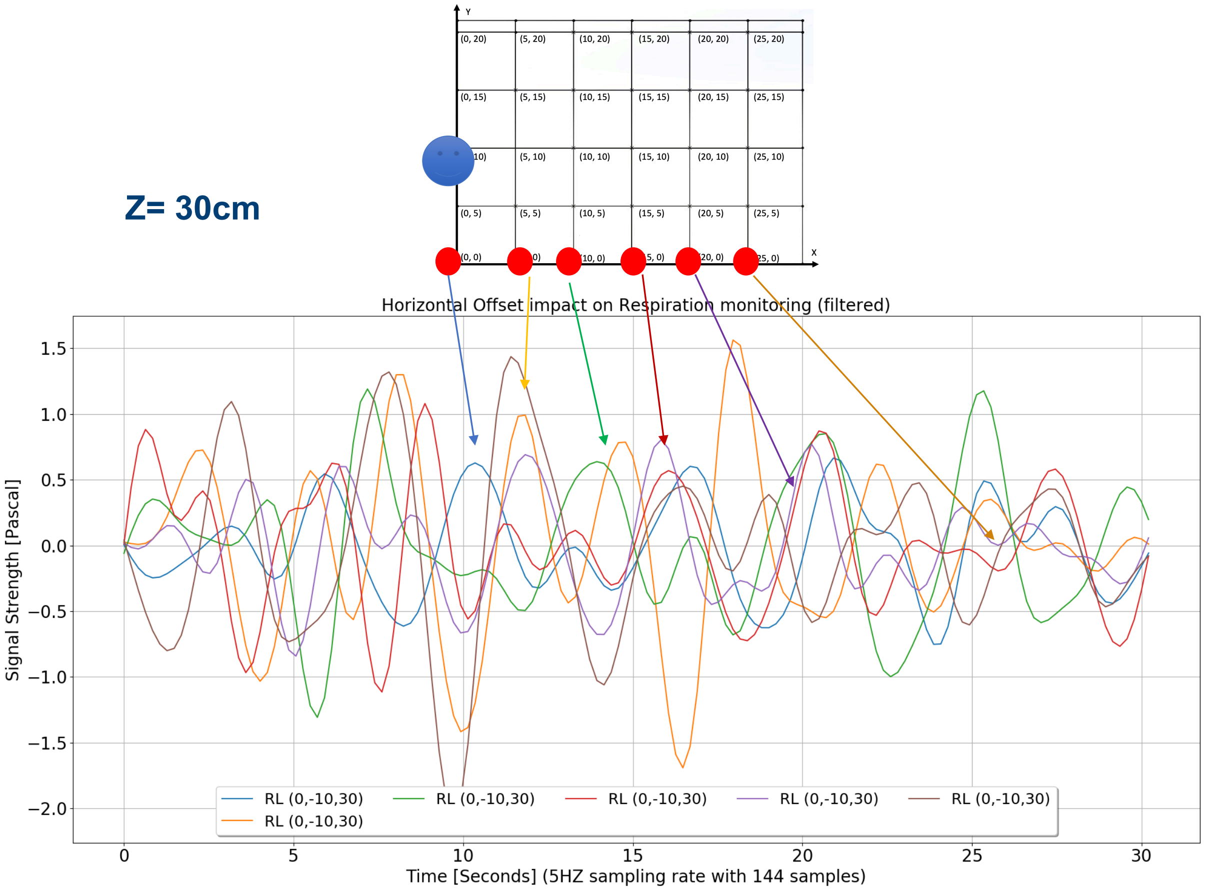

FIGURE S1. Signal strength distribution with 30 cm Z offset, -10 cm Y offset and 0-25 cm X offset.

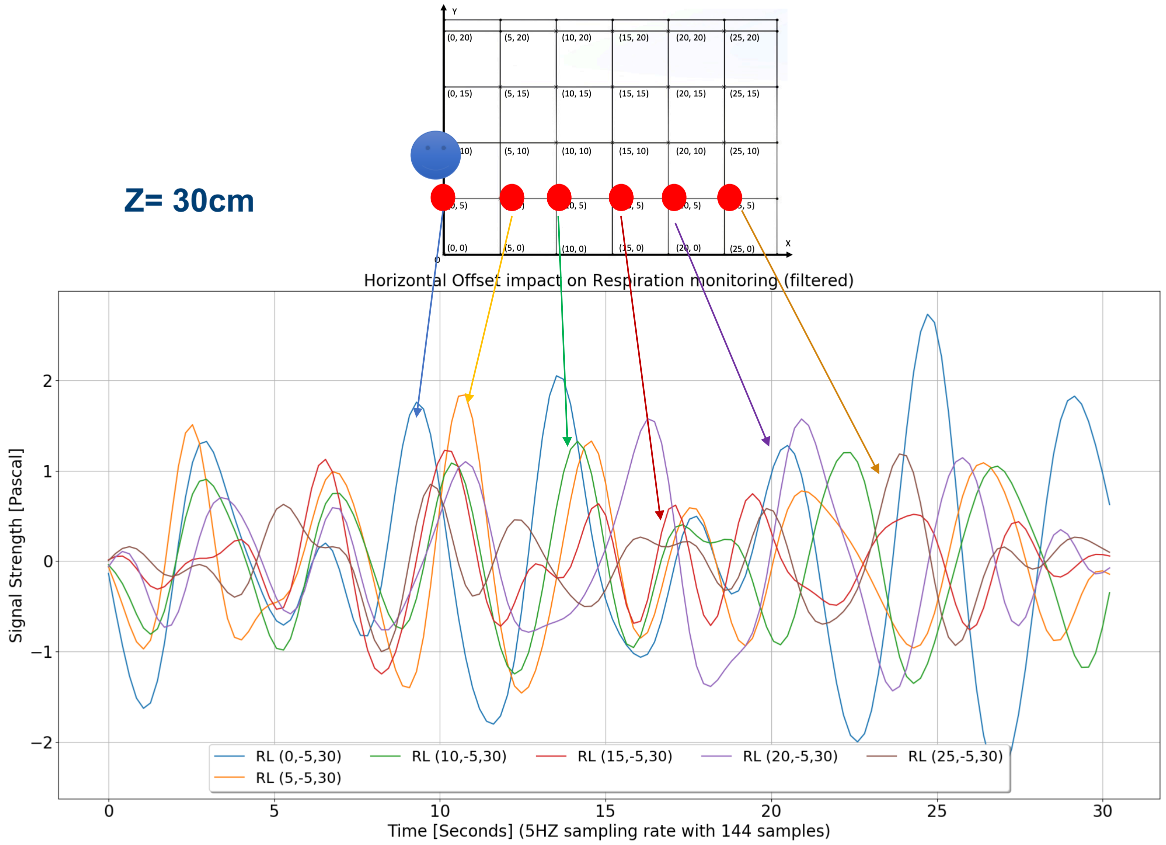

FIGURE S2. Signal strength distribution with 30 cm Z offset, -5 cm Y offset and 0-25 cm X offset.

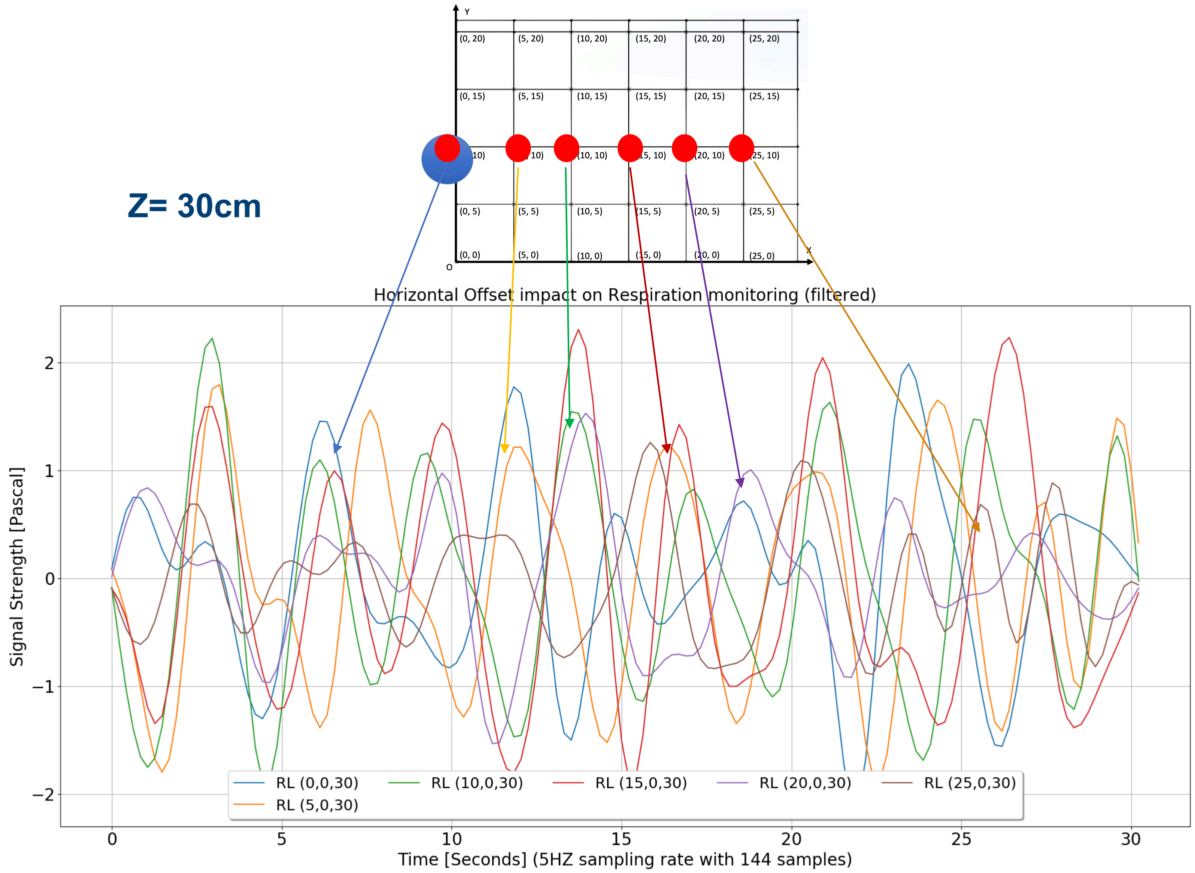

FIGURE S3. Signal strength distribution with 30 cm Z offset, 0 cm Y offset and 0-25 cm X offset.

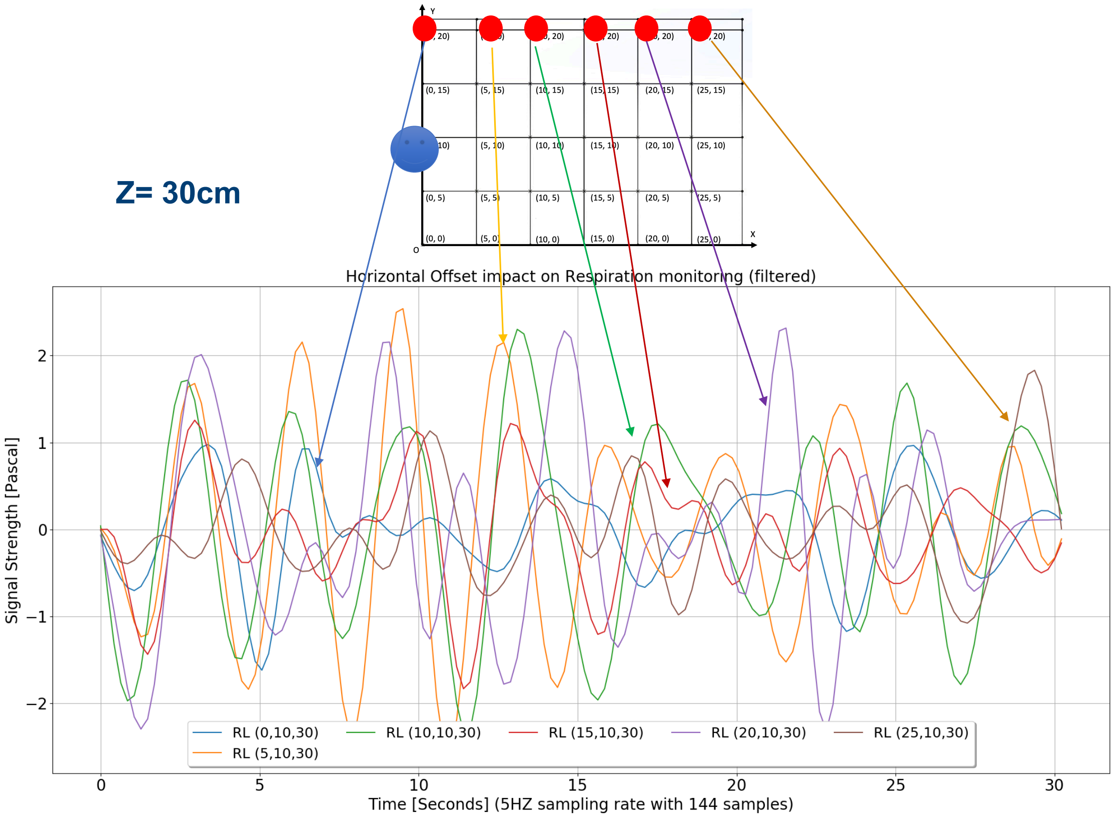

FIGURE S4. **Signal strength distribution with 30 cm Z offset, 5 cm Y offset and 0-25 cm X offset.**

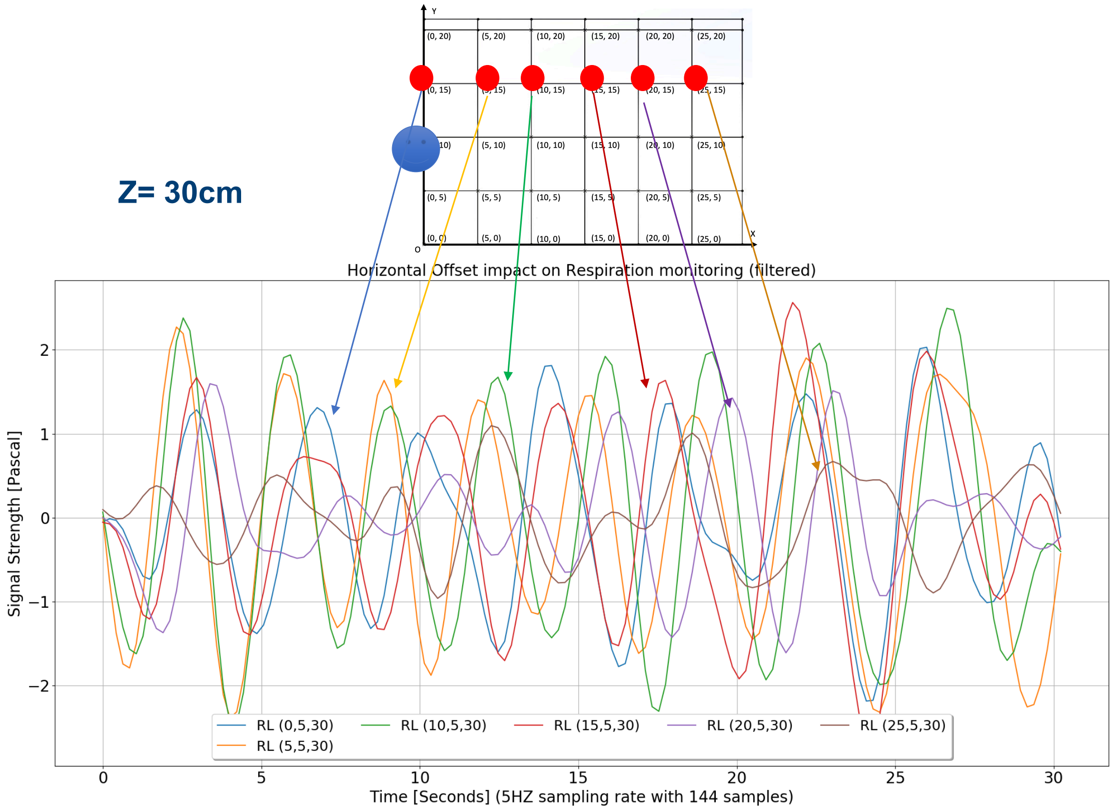

FIGURE S5. **Signal strength distribution with 30 cm Z offset, 10 cm Y offset and 0-25 cm X offset.**

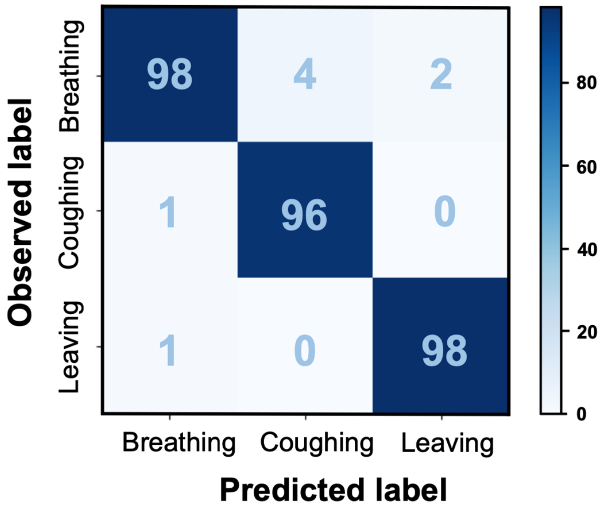

FIGURE S6. **Confusion matrix of coughing detection.**

TABLE II. ALGORITHM FOR DUPLICATE POINTS REDUCTION

```
Input: Peak, valley points matrix [P, V] and Respiration matrix [X]
Output: Crossing peak-valley points location matrix [P_c, V_c]
i = 0; j = 0; S = min(P[0], V[0]);
while i < len(P) and j < len(V) do
    if P[i] < V[j]:
        if S == 'valley':
            S = 'peak'; I +=1;
        else:
            if abs (X_{V_{j-1}} − X_{P_i}) > abs (X_{V_{j-1}} − X_{P_{i-1}})
                Del_Peak.append(i-1)
            else:
                Del_Peak.append(i)
    else:
        if S == 'peak':
            S = 'valley'; j += 1;
        else:
            if abs (X_{P_{i-1}} − X_{V_j}) > abs (X_{P_{i-1}} − X_{V_{j-1}}):
                Del_Peak.append(j-1)
            else:
                Del_Peak.append(j-1)
Pc = P [m ∉ Del_Peak for m in P]
Vc = V [n ∉ Del_Valley for n in V];
return [P_c, V_c]
```

TABLE I. A COMPARISON OF SPECIFICITY OF CURRENT RESPIRATION MONITORING SYSTEMS

Method	Specificity	System sensitivity	Payload convenience	Expense Cost	Privacy
Accelerometer [1]	82.34%	Activity movements	Wearable	Low	Yes
Radar [2]	80%	Environmental signal	Ambient	High	Yes
WiFi [3]	89.78%	Electronics signal	Ambient	Low	Yes
Acoustic sensor [4]	96.58%	Spatial distance	Ambient	Low	No
Stretchable sensor [5]	-	Activity movements	Wearable	High	Yes
Thermal imaging [6]	99.9%	Spatial distance	Ambient	Low	No
Thermistor [7]	69.27%	Breathing strength	Invasive	Low	Yes
Capnometry [8]	12%	Breathing depth	Wearable	High	Yes
Our system	98.98%	Spatial distance	Ambient	low	Yes

Reference

[1] D. Phan, S. Bonnet, R. Guillemaud, E. Castelli, and N. P. Thi, "Estimation of respiratory waveform and heart rate using an accelerometer," in *2008 30th Annual International Conference of the IEEE Engineering in Medicine and Biology Society*, 2008: IEEE, pp. 4916-4919.

[2] Y. Xiao, C. Li, and J. Lin, "A portable noncontact heartbeat and respiration monitoring system using 5-GHz radar," *IEEE Sensors Journal,* vol. 7, no. 7, pp. 1042-1043, 2007.

[3] X. Liu, J. Cao, S. Tang, J. Wen, and P. Guo, "Contactless respiration monitoring via off-the-shelf WiFi devices," *IEEE Transactions on Mobile Computing,* vol. 15, no. 10, pp. 2466-2479, 2015.

[4] C. Liu, J. Xiong, L. Cai, L. Feng, X. Chen, and D. Fang, "Beyond respiration: Contactless sleep sound-activity recognition using RF signals," *Proceedings of the ACM on Interactive, Mobile, Wearable and Ubiquitous Technologies,* vol. 3, no. 3, pp. 1-22, 2019.

[5] A. Bates, M. J. Ling, J. Mann, and D. K. Arvind, "Respiratory rate and flow waveform estimation from tri-axial accelerometer data," in *2010 International Conference on Body Sensor Networks*, 2010: IEEE, pp. 144-150.

[6] A. H. Alkali, R. Saatchi, H. Elphick, and D. Burke, "Thermal image processing for real-time non-contact respiration rate monitoring," *IET Circuits, Devices & Systems,* vol. 11, no. 2, pp. 142-148, 2017.

[7] A. Sabil *et al.*, "Comparison of apnea detection using oronasal thermal airflow sensor, nasal pressure transducer, respiratory inductance plethysmography and tracheal sound sensor," *Journal of Clinical Sleep Medicine,* vol. 15, no. 2, pp. 285-292, 2019.

[8] B. G. Goudra, L. C. Penugonda, R. M. Speck, and A. C. Sinha, "Comparison of acoustic respiration rate, impedance pneumography and capnometry monitors for respiration rate accuracy and apnea detection during GI endoscopy anesthesia," 2013.
